# Sperm Proteomics: Road to Male Fertility and Contraception

**DOI:** 10.1155/2013/360986

**Published:** 2013-12-01

**Authors:** Md Saidur Rahman, June-Sub Lee, Woo-Sung Kwon, Myung-Geol Pang

**Affiliations:** Department of Animal Science and Technology, School of Bioresource and Bioscience, Chung-Ang University, 4726 Seodong-daero, Anseong, Gyeonggi-Do 456-756, Republic of Korea

## Abstract

Spermatozoa are highly specialized cells that can be easily obtained and purified. Mature spermatozoa are transcriptionally and translationally inactive and incapable of protein synthesis. In addition, spermatozoa contain relatively higher amounts of membrane proteins compared to other cells; therefore, they are very suitable for proteomic studies. Recently, the application of proteomic approaches such as the two-dimensional polyacrylamide gel electrophoresis, mass spectrometry, and differential in-gel electrophoresis has identified several sperm-specific proteins. These findings have provided a further understanding of protein functions involved in different sperm processes as well as of the differentiation of normal state from an abnormal one. In addition, studies on the sperm proteome have demonstrated the importance of spermatozoal posttranslational modifications and their ability to induce physiological changes responsible for fertilization. Large-scale proteomic studies to identify hundreds to thousands of sperm proteins will ultimately result in the development of novel biomarkers that may help to detect fertility, the state of complete contraception, and beyond. Eventually, these protein biomarkers will allow for a better diagnosis of sperm dysfunctions and aid in drug development. This paper reviews the recent scientific publications available from the PubMed database to address sperm proteomics and its potential application to characterize male fertility and contraception.

## 1. Introduction

Spermatozoa are highly specialized cells with specific metabolic pathways compartmentalized in different regions. Unlike other body cells, spermatozoa are exceptionally differentiated and show large variations in their genetic, cellular, and chromatin structures, which allow them to control fertility/infertility, embryo development, and heredity. Physiologically normal spermatozoa are required for successful fertilization, whereas suboptimal sperm quality with abnormal motility, morphology, concentration, DNA fragmentation, chromatin stability, and genetic composition has been directly linked to male factors of infertility [1–3]. However, our current understanding of spermatozoa, including their different physiological and pathological aspects, is still limited and not well defined. The human genome has been sequenced more than 10 years ago with a large readily accessible database, which is considered the starting point for the understanding of how a tissue-specific cell functions on the molecular level in health and disease conditions [4]. However, without considering proteomics, DNA sequence data alone answer very few or no questions about the level of protein expression. Therefore, to explore the biological display of the human genome, the physiological functions of each protein should be understood first [4, 5].

Proteomics involves the comprehensive study of proteins with their particular structural and functional aspects [6]. Recent studies of spermatozoa from the proteomic point of view have allowed the identification of different proteins in spermatozoa that are responsible for the regulation of normal/defective sperm functions. Mature spermatozoa are inactive with regard to transcription, translation, and protein synthesis [1] and are, therefore, very suitable for proteomic analysis [7]. As such, proteomics has the potential to transform our understanding of how mature sperm cells work. In addition, it is important to note that a sperm cell contributes about half of its nuclear genetic material to the successive diploid offspring during fertilization; thus, examining the genetic composition of spermatozoa might also provide beneficial insights into congenital disorders.

At present, numerous proteomics techniques, including two-dimensional (2D) polyacrylamide gel electrophoresis, mass spectrometry (MS), and differential in-gel electrophoresis, are widely used to identify particular sperm-specific proteins. These approaches have offered a concise but deep understanding of different functional aspects of sperm proteins, for example, motility, capacitation, acrosomal reaction, fertilization, and posttranslational modifications such as phosphorylation, glycosylation, proteolytic cleavages, and mutations. In addition, proteomic research has also provided a new horizon to study different functional states of spermatozoa, for example, normal versus malformed, immature versus mature, capacitated versus incapacitated, low versus high sperm count, low versus high fertility, and normal/highly fertile versus state of complete contraception [1, 8–10], which are all necessary and important to identify a suitable biomarker and to select the desired sire. Furthermore, design and construction of agonistic/antagonistic approaches using commercially synthesized, purified chemicals with regard to differentially expressed proteins of spermatozoa might be helpful to develop fertility enhancers, new treatments for infertility, and contraception pills. The present work reviews the latest articles published by other laboratories as well as our research team on the proteomics aspect of spermatozoa and their potential implications to assist fertility or to develop a male contraceptive strategy.

## 2. Biology of the Sperm Cell

**Figure 1:**
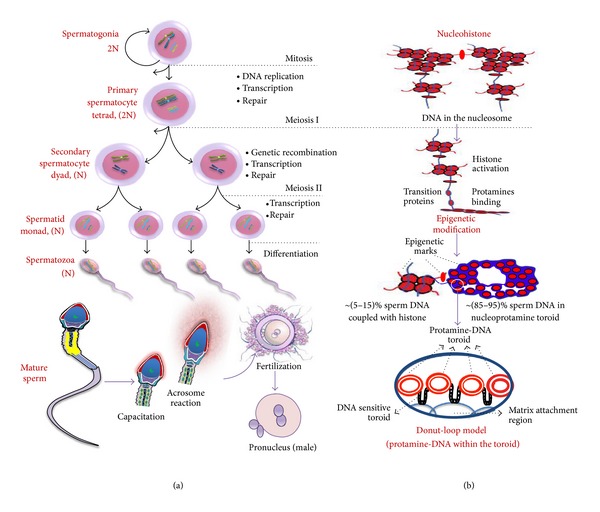
Schematic diagram showing the cellular, genetic, and chromatin changes during spermatogenesis. (a) The figure represents the cellular changes during spermatogenesis coupled with its genetic modifications. Following spermatogenesis, the male primordial germ cells, spermatogonia, first differentiate to primary spermatocytes and undergo genetic recombination to produce haploid round spermatids. The round spermatids then participate in another differentiation process to produce the mature spermatozoa. For successful fertilization, mammalian spermatozoa undergo first capacitation and then the acrosome reaction, which collectively allows spermatozoa to penetrate the zona pellucida and to fuse with the oocyte plasma membrane both *in vivo* and *in vitro*. (b) The figure shows chromatin changes that cause the transition of the mammalian nucleohistone to nucleoprotamine [17, 18]. In addition, the Donut-Loop model has been inserted at the end to represent the internal structure of the protamine-DNA fibers inside the toroid [17, 18]. The red color indicates histones and the blue color indicates DNA.

Spermatozoa are mature male gametes that are produced through a unique process called spermatogenesis. In mammals, spermatogenesis occurs in the male testes and epididymis in a stepwise fashion that is highly dependent on optimal conditions for the process to work correctly. Spermatozoa undergo morphological, biochemical, and physiological modifications in the testes and epididymis [11]. However, the complete differentiation process largely depends on changes at the genetic, cellular, and chromatin level [11–14]. Cellular adjustment that occurs during spermiogenesis includes nuclear condensation, movement of the nucleus to the periphery of the cell, and formation of the acrosome and flagella (depicted in Figure 1). To attain successful fertilization both *in vitro* and *in vivo*, mammalian spermatozoa must undergo capacitation followed by the acrosome reaction, which are important processes that finally allow the mature sperm cell to penetrate the zona pellucida and to fuse with the oocyte plasma membrane during fertilization and early embryonic development [15] (see Figure 1(a)). In contrast, the entire process of spermiogenesis results in significant changes in the sperm chromatin structure and DNA strand breaks to allow the accompanying change in DNA topology (see Figure 1(b)). Both *in vitro* and *in vivo* evidence suggests that the DNA-binding and condensing activities of a set of basic nuclear “transition proteins” is critical to the integrity of the entire chromatin remodeling process [16].

## 3. Background of Sperm Proteomic Research

Comprehensive identification and quantification of various proteins expressed in cells and tissues offer important and fascinating insights into the dynamics of cellular functions and differentiation. The novel approaches of characterizing proteins by means of both qualitative and quantitative analyses are known as proteomics. Proteins carry out a wide range of functions, from forming structural materials to catalyzing chemical reactions; thus, knowing exactly which proteins are in sperm cells is a major step forward in understanding. The so-called proteome of spermatozoa contains everything the sperm needs to survive and function correctly. The study of sperm proteins was in fact initiated with the remarkable finding by Friedrich Miescher in 1874. Miescher was able to identify the basic component of salmon spermatozoa called “protamine” that was coupled with nuclein, which was known as DNA afterward. Oliver and Dixon [17] and Balhorn [18] noted that the protamines initially isolated by Miescher are the most abundant sperm nuclear proteins in many animal species, as well as the initial proteins identified in the spermatozoal nucleus. However, the founders of sperm proteomics study were Naaby-Hansen and his colleagues from Virginia, and their elegant studies addressed the first human's sperm protein database of 1400 spots [19]. With the recent development of modern proteomic approaches such as the application of narrow-range isoelectric focusing strips and multiple 2D gels, this number might be much higher than that previously reported [20–22].

## 4. Basic Techniques of Sperm Proteomic Research

In order to understand how sperm proteins can be identified and characterized, the fundamental process of proteomic analysis will be addressed in brief. After collection and liquefaction of a semen sample, it can be used as it is or it can be subjected to additional processing for purification of spermatozoa. The ejaculate comprises various cellular components, for example, seminal plasma, secretions from the accessory sex gland, blood cells, and epithelial cells. Therefore, during sperm proteomic analysis, contamination of seminal plasma proteins might be the reason for the substantial differences observed between samples from the same donors [23, 24]. To minimize this type of contamination, the direct swim-up method and the density gradient method (using Percoll) have been suggested previously for sperm isolation in humans [23, 24]. However, the Percoll method demonstrated much better reproducibility than the swim-up method [23]. Recently, different density gradient methods have been applied to isolate spermatozoa from both human and animal subjects. For example, a discontinuous density gradient of 90%−45% Percoll was used to remove extender, debris, seminal plasma, and dead spermatozoa in bovine semen [24, 25]. A 35% PureSperm solution was used to separate caput and cauda epididymal sperm in mice [26]. In swine, a two-layer isotonic Percoll gradient (90%−40%) was used to purify epididymal sperm [27], whereas 80%−55% Percoll was used to separate spermatozoa from the ejaculate [28]. On the basis of the aforementioned studies, it is important to select purification methods for sperm cells that are suitable for the species studied.

The basic techniques used in proteomics research largely focus on illuminating the structure and conformation, purifying, and measuring the concentration of sperm proteins. The proteomic techniques that are used for the structural analysis of sperm proteins include MS and electrophoresis [26, 27], nuclear magnetic resonance spectroscopy [29, 30], and X-ray crystallography [31, 32]. However, liquid chromatography (LC) [33, 34] and the recently developed microfluidic separation technique [35, 36] are widely used for the purification of proteins from spermatozoa or other samples. In addition, western blot [37, 38] and chemical microarrays [39] are used to determine the concentration/density of a particular protein in sperm cells by using the corresponding antibody of the protein of interest. Furthermore, various stains such as Coomassie brilliant blue and silver stain are used to identify the positions of individual proteins within the gel, which appear as spots or smudges. Data from the structural analysis can be used to identify the functions of various sperm proteins, by comparison with similar proteins in the sperm with known functions. However, this process generally requires a large protein conformation as well as available functional databases, sophisticated software to analyze the data, and an algorithm platform.

**Figure 2:**
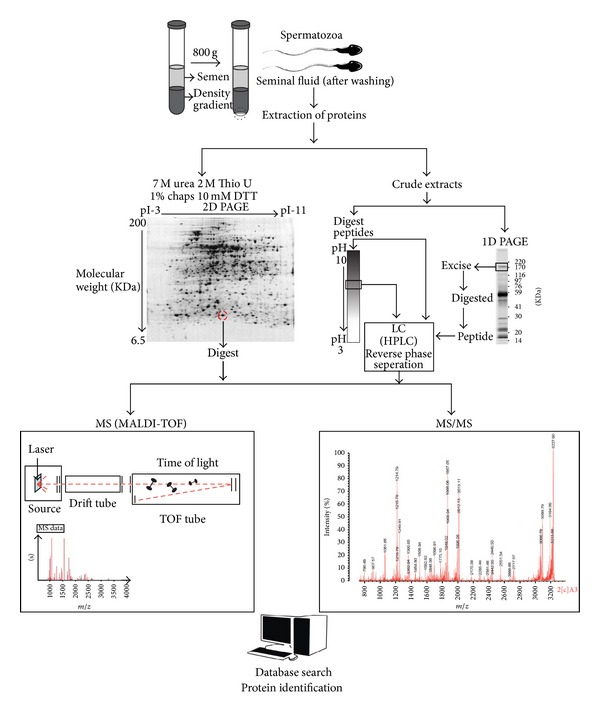
Schematic diagram showing the extraction and analysis of proteins from sperm cells. After collection and liquefaction of a whole semen sample, it can be used as it is or it can be subjected to density gradient centrifugation to separate mature and immature sperm populations. The recovered spermatozoa can be extracted and purified, after which the protein concentration can be determined in order to allow for an equal protein-loading gradient on electrophoretic gels. The further analysis of the sperm proteome is done from an aliquot of the sperm cells by using two-dimensional polyacrylamide gel electrophoresis (2D-PAGE) followed by excision of protein spots from the 2D-PAGE gels and matrix-assisted laser desorption/ionization-mass spectroscopy (MALDI-MS) or tandem MS (MS/MS) identification. Alternatively, the initial protein extract can be digested into peptides, which can be separated via liquid chromatography, followed by MS/MS analysis.

However, the most frequently used extraction and analysis process of proteins from spermatozoa involves 2D gel electrophoresis followed by excision of protein spots from the 2D gel and matrix-assisted laser desorption/ionization-MS (MALDI-MS) or tandem MS (MS/MS) identification [20, 40, 41]. Alternatively, the initial protein extract is digested into peptides, separated through LC, and analyzed by MS/MS (see Figure 2) [42, 43].

## 5. Proteomic Approach to Define Male Fertility

Recently, proteomic tools have gained a unique position because of their significant contribution in protein research. As a major technique, such proteomic approaches are largely applied to identify proteins that regulate the biological functions of a cell. In the field of sperm cell biology, the identification of a wide range of proteins provides information to uncover the regulatory mechanism of male fertility as well as infertility for which the reason is unknown or poorly understood. The current section reviews the value of proteomics in male fertility by using the recently available scientific information.

Reports show that the male factors account for approximately 50% of all infertility in human and bovine when artificial insemination has been used [1]; however, the underlying molecular mechanisms are still not understood [44]. Over the past few decades, the traditionally available methods to detect male fertility include assessments of sperm morphology [45], motility [46], mucus penetration test (cervical) [47], membrane intactness [48], the acrosome reaction [49], ATP concentration in semen [50], and sperm-zona binding capability [51]; however, their clinical value is always debated [52]. Therefore, the identification of marker proteins of fertility might be more appropriate to determine the male potentiality for successful reproduction.

**Figure 3:**
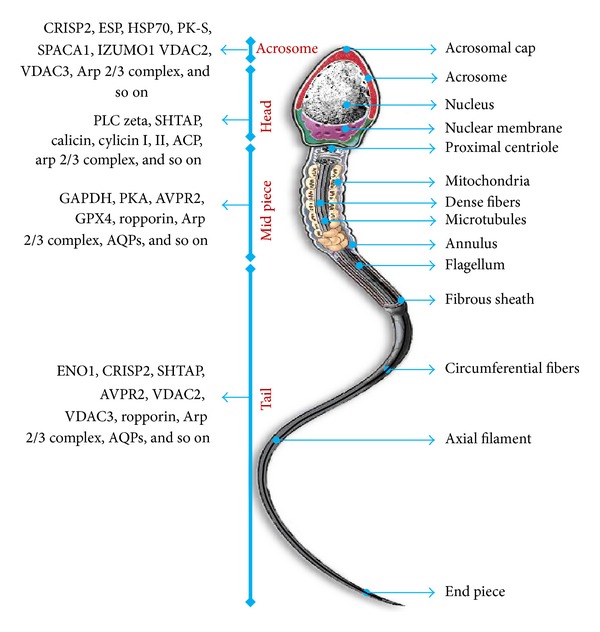
Diagram showing a mature human spermatozoon with its different parts. Previously published studies were used as a reference to summarize the list of proteins localized in different regions of the spermatozoon by immunolocalization. Proteins localized in acrosome, head, midpiece, and tail are mainly involved in capacitation and acrosomal reaction, spermatozoa-oocyte interaction (zona binding), energy production and metabolism, and motility and metabolism, respectively. The localization of a single protein at multiple sites in the sperm cell indicates its potential involvement in different physiological processes of the spermatozoon. The listed proteins include protein kinase A (PKA), phospholipase C zeta (PLC zeta), sperm head- and tail-associated protein (SHTAP), calicin, cylicins I and II, actin-capping proteins (ACP), enolase 1 (ENO1), cysteine-rich secretory protein 2 (CRISP2), arginine vasopressin receptor (AVPR), voltage-dependent anion channel (VDAC), glyceraldehyde 3-phosphate dehydrogenase (GAPDH), glutathione peroxidase 4 (GPX4), equatorial segment protein (ESP), heat shock protein 70 (HSP70), novel pyruvate kinase (PK-S), sperm acrosome membrane-associated protein 1 (SPACA1), izumo sperm-egg fusion 1 (IZUMO1), aquaporin 7 (AQPs), and the actin-related protein 2/3 complex (Arp2/3 complex).

In recent work from our laboratory, we have identified differential protein expression profiles from high- and low-fertility bulls in order to characterize high-fertility bull spermatozoa [1]. In this study, the lower limit of the higher fertility was defined as >70% nonreturn rate. Database searching helped to identify proteins that are highly expressed in fertile bull spermatozoa. The identified proteins involved enolase 1, ATP synthase H^+^, transporting mitochondrial F1 complex beta subunit, apoptosis-stimulating of p53 protein 2, alpha-2-HS-glycoprotein, and phospholipid hydroperoxide glutathione peroxide; these proteins primarily regulate metabolism, posttranslational modification, and motility of spermatozoa. However, 3 proteins of low-fertility bulls were highly represented, that is, voltage dependent anion channel 2 (VDAC2), ropporin-1, and ubiquinol-cytochrome-c reductase complex core protein 2. These findings are in accordance with the results of Liu et al. [53], who reported that VDAC2 may induce infertility with idiopathic asthenozoospermia. However, several reports have shown that ubiquinol-cytochrome-c reductase complex core protein 2 is associated with oxidative stress and ROS generation in mitochondria [54, 55]; in spermatozoa, both events play a paradoxical role in the regulation of fertility. However, we were unable to represent a mechanism by which ropporin-1 correlates with low fertility. Finally, we demonstrated that enolase 1, VDAC2, and ubiquinol-cytochrome-c reductase complex core protein 2 were significantly correlated with individual fertility and essentially represent the true fertility markers for the respective bull. Therefore, a similar study will certainly be helpful to construct and design a new biomarker to identify fertile individuals. Gaviraghi et al. [56] have reported that the candidate protein of a high-fertility bull is involved in sperm-egg interactions and cell cycle regulation. In their study, 2D electrophoresis coupled with MALDI-MS techniques was used to identify the differentially expressed proteins. Notably, Peddinti et al. [25] employed the differential detergent fractionation multidimensional protein identification technology and identified 125 putative biomarkers of fertility. In particular, they found that high-fertility bull spermatozoa contained higher levels of proteins that are involved in energy metabolism, cell communication, spermatogenesis, and cell motility. In addition, similar methods together with 2D electrophoresis and LC-MS/MS were considered by D'Amours et al. [57] to identify high-fertile Holstein bulls. However, these researchers were able to identify only 2 proteins (adenylate kinase isoenzyme 1 and phosphatidylethanolamine-binding protein 1) that are mainly expressed in fertile bull spermatozoa and that can explain 64% (*P* < 0.001) of the fertility scores of the respective bull. Therefore, it is important to note that a major variation in protein identification always exists in these studies. The most straightforward explanation for this variation might be to consider the different breeds of animals as well as the fact that in most of these studies, 2D electrophoresis analysis was conducted with pooled samples. In addition, contamination of seminal plasma proteins might be another reason for the substantial differences observed between samples from the same donors. Therefore, intensive studies on individual proteins and their contribution to reproduction should be conducted to address these open questions. Furthermore, immunolocalization and expression patterns of such proteins should also be considered as they can readily be used to obtain some information about their function (see Figure 3).

For successful fertilization, spermatozoa should first undergo capacitation [58–60] and then the acrosome reaction [15], which is the common sequence of biochemical and physiological modifications facilitating the sperm cell to penetrate the zona pellucida and to fuse with the oocyte plasma membrane. A capacitation-induced alteration or acrosome reaction of the sperm proteome can, therefore, be a consequence of posttranslational modifications of pre-existing proteins or of the expression of new proteins, or of both. In any case, proteomic approaches are mandatory for the elucidation of the molecular processes underlying capacitation and acrosomal reaction, both of which should be described in more detail. For a better understanding of the fertilizing capability of males, attempts have been made in order to identify spermatozoa that differentially express proteins related to such fundamentally important events. In Table 1, a brief summary is given of recent animal studies where proteomic approaches have been utilized towards addressing male fertility.

Since 10 years, relatively few studies in the literature have examined the proteome of human spermatozoa in order to define the fertile man; however, recently, the number of similar studies on human spermatozoa has been rapidly increasing. Pixton et al. [41] utilized a man who experienced failed fertilization at *in vitro *fertilization (patient) and different fertile controls. They reported that although the proteome variation among the fertile donors was very low, they were able to identify 20 proteins that were differentially expressed between fertile and non-fertile patients. However, a main drawback of this study is that they considered only 1 patient. Therefore, consideration of a wider range of samples with available field data (fertilizing capability, nonreturn rate in animals) might be helpful to identify particular differences in the protein expression that can represent the fertile individual more accurately. Eventually, such studies will enable further elucidation of the underlining molecular mechanisms and might shed additional light on key sperm proteins involved in infertility.

Apart from the fact that there are few other studies that attempted to characterize the sperm plasma membrane [71], spermatozoa-specific processes such as calcium-binding ability and capacitation [72] also provide important information on the fertility of human as well as animal species. Although it was almost more than 15 years ago that tyrosine phosphorylation was identified as an indicator of capacitation and it is known to play an essential role in fertilization, the role of the proteins and their associated sequence of activation is still a matter of question. Only a small number of candidate proteins for this important event of fertilization have been identified to date [73]. Reports demonstrated that the phospholipid hydroperoxide glutathione peroxidase in spermatozoa of hamster is phosphorylated during tyrosine phosphorylation following capacitation [74]. However, it is still a long way to reveal the whole picture of human sperm physiology, especially from the proteomic point of view. We summarized some proteomics studies on human spermatozoa where attempts have been made to identify male fertility (Table 2).

## 6. Sperm Proteomic versus Male Contraception

The world population continues to increase more rapidly, even after female contraception has been significantly emphasized. This pressure of increased population has caused various environmental hazards, global warming, hunger, and diseases. Consequently, researchers are now looking for suitable ways to control the birth rate of humans as well as of domestic animals, especially pets. Reports have shown that about half of all human pregnancies are unwanted or unexpected [82]. Therefore, it becomes obvious to consider the participation of men in contraceptive programs. The most available male contraception methods involve the use of condoms and vasectomy. The efficacy of such methods is quite satisfactory; however, their popularity is argued. At present, few hormonal methods (injected, implanted, or taken orally) are also used for male contraception, which is believed to play an important role to serve this purpose [10]. Although these methods have some drawbacks as they cause minor but significant effects on a number of non-reproductive parameters, some of them are used with considerable popularity. However, frequently arising questions against the use of such methods include their potential efficacy for providing a state of contraception. Therefore, it is important to determine the efficacy of such methods in addition to searching for new methods that could potentially be used to serve this purpose while providing higher protection. The present section will review the application of proteomic approaches to confirm the state of complete contraception by using existing contraception methods as well as to establish new methods for male contraception.

In an advance headed for a long-sought new male contraceptive, researchers have attempted to identify key proteins in men that could effectively suppress the production of sperm, which might become a new target for a potential male birth control pill. In male hormonal contraception, the administration of supraphysiologic doses of testosterone (TU) has been shown to significantly reduce circulating gonadotropins and induce severe oligozoospermia or azoospermia [82]. However, supraphysiologic doses of TU are unable to suppress spermatogenesis uniformly and are associated with weight gain and suppression of high-density lipoprotein cholesterol [83, 84]. Exogenous progestins are also known to suppress circulating gonadotropins and might synergistically suppress spermatogenesis when combined with exogenous TU. Therefore, various combination regimens of TU plus a progestin such as levonorgestrel and cyproterone acetate have been reported as potential hormonal contraceptives. An elegant study conducted by Cui et al. [10] reported that TU combined with levonorgestrel was able to change 31 sperm proteins compared to only 13 proteins that changed in men given only testosterone. In addition, this research group noted that treatment of men with TU alone or TU + levonorgestrel effectively suppressed spermatogenesis through multiple signaling pathways by altering the expression of different proteins. Therefore, it is tempting to hypothesize that the identified proteins could serve as both targets for new male contraceptives as well as medications for treating men that experience infertility. However, repeated trials are necessary in order to represent a specific group of proteins to address the state of complete contraception. In addition, Kwon et al. [38] have reported that exposure of mouse spermatozoa to deamino [Cys 1, d-ArgS] vasopressin, an arginine vasopressin receptor-2 agonist, significantly decreased sperm motility, intracellular pH, and phospho-protein kinase A substrates and increased Ca^2+^ concentration. This research helped to summarize that a higher level of arginine vasopressin, a neurohypophysial hormone, has a damaging effect on overall sperm function. Therefore, such study might also be important to the progress of diagnostic tests to access male potentiality for fertilization.

Nonhormonal methods, including the use of compounds that target sperm functionality, might also be attractive because of their theoretical promise of specificity for the reproductive tract. The study of sperm proteomics continues to identify possible targets for this approach. Such nonhormonal agents will likely enter clinical trials in the near future. Recently, we have reported that blocking VDAC2 and VDAC3 proteins with 4,4′-diisothiocyanostilbene-2,2′-disulfonic acid significantly decreased sperm motility, viability, acrosome reaction, capacitation, tyrosine phosphorylation, fertilization, and embryo development and proposed that VDACs are essential for successful reproduction [37]. This study indicated that the abnormal functioning of VDACs negatively affects sperm functions and that VDACs act as one of the key regulators for the fertilizing ability of spermatozoa. More recently, Shukla et al. [85] reported that nutlin-3a inhibits the functions of ubiquinol-cytochrome-c reductase core protein 2 in spermatozoa during capacitation, resulting ultimately in decreased male fertility. Therefore, it is suggested that similar studies should be conducted to identify particular proteins that could play pivotal roles in male fertility/infertility. Moreover, a wide range of studies targeting a single protein with a high number of repetitions can improve its application in clinical conditions.

## 7. Conclusion and Future Directions of Sperm Proteomic Research Targeting Male Fertility and Contraception

The belief in the modern world is that spermatozoa provide a potential source of study to investigate the numerous outstanding findings that control reproduction in general. Proteomics represents an extraordinary tool for evaluating the molecular mechanisms regulating sperm function, for shedding light on our understanding of the male fertility/infertility, and for characterizing the state of complete contraception. It can be considered to address spermatozoal posttranslational modifications as well as to elucidate the molecular basis of defective sperm functions with implications for our ability to both diagnose and treat this condition. Therefore, the identified proteins should be rigorously tested to determine whether they have any clinical value for fertility/infertility evaluation. Simultaneously, factors that can affect proteomics should be considered, and all of these must be taken into account when translating discovery proteomics into clinical trials and practice.

Several databases of sperm proteins are now acting as an important source of knowledge. Catalogues of proteins derived from proteomic approaches are valuable as they represent the group of proteins that can be exposed in a particular physiological/pathological condition more specifically; however, the functions of each protein are still unknown [40, 80, 86]. Some proteins exhibit various functions; they may play different roles in the whole organism, but may have a specific function in spermatozoa. Therefore, intensive studies on individual proteins (i.e., agonistic and antagonist chemicals and knockout and knock-in models) and their contribution to reproduction should be undertaken to address these open questions.

Comparison of data from the proteome and transcriptome also provides a more reliable basis to evaluate the exact mechanisms of the respective protein that controls fertility/infertility or contraception. Taken together, it may provide new avenues for the management of male reproduction in general and novel targets for expected reproductive outcomes.

## Figures and Tables

**Table 1:** Summary of major proteomic studies to define the fertile male (animal models).

Sample	Proteomic technique	Study outcome	Authors
Mouse spermatozoa	LC-MS/MS	Identification of 52 proteins following capacitation	Baker et al. [61]
Boar spermatozoa	LC-MS	Identification of proteins from the sperm surface that are responsible for binding of the sperm to the oocyte	Belleannee et al. [27]
Bull spermatozoa	LC-MS/MS	Identification of 131 proteins to differentiate normal and pyriform sperm	Shojaei Saadi et al. [62]
Bull spermatozoa (Holstein)	LC-MS/MS	Identification of proteins of impaired sperm function due to elevated testicular temperature, with important implications for fertility predictions	Newton et al. [63]
Accessory sex gland fluid (Holstein)	LC-MS/MS	Increased expression of bovine seminal plasma protein identified in high-fertility bulls	Moura et al. [64]
Accessory sex gland (dairy bull)	LC-MS/MS	Identification of isoforms of osteopontin and phospholipase A2 (PLA2) in high-fertility bulls	Moura et al. [65]
Mouse spermatozoa	MS/MS	Identification of 299 testis-specific proteins and detection of 155 novel proteins involved in spermiogenesis	Guo et al. [66]
Mouse spermatozoa	MS/MS	Identification of 100 proteins that are expressed on mature sperm at the site of sperm-oocyte interactions	Stein et al. [67]
Rat spermatozoa	LC-MS/MS	Identification of 5123 peptides from rat spermatozoa	Baker et al. [68]
Mouse spermatozoa	MS/MS	Identification of capacitation-associated changes in the phosphorylation status (55 *in vivo* sites) of mouse sperm proteins	Piatt et al. [69]
Mouse spermatozoa	PMF, LC-MS/MS	Identification of 100 proteins of capacitation	Nixon et al. [70]

LC-MS/MS: liquid chromatography-tandem mass spectrometry; LC-MS: liquid chromatography-mass spectrometry; MS/MS: tandem mass spectrometry; PMF: peptide mass fingerprints.

**Table 2:** Proteomic studies on human spermatozoa to access male fertility.

Authors	Proteomic technique	Study outcome
Zhao et al. [75]	MALDI-TOF	Identification of 10 differentially expressed proteins in asthenozoospermic patients compared with those of normozoospermic donors
Martínez-Heredia et al. [40]	MALDI-TOF	Identification of 131 proteins to provide a reference 2D map of mature human sperm spermatozoa
Johnston et al. [76]	LC-MS/MS	Identification of 1760 proteins that comprise human sperm
Domagala et al. [77]	LC-MS	Identification of 35 proteins as sperm immunogenic antigens
Secciani et al. [78]	MS/MS	Identification of protein profiles of capacitated versus ejaculated spermatozoa
Li et al. [20]	MALDI-MS	Establishment of a high-resolution 2D map of human sperm proteins by identifying 16 proteins
Chan et al. [79]	MADLI-TOF	Identification of 12 proteins to address altered protein phosphorylation and aberrant sperm motility
Baker et al. [42]	LC-MS/MS	Identification of 1056 gene products from human sperm populations
Lefièvre et al. [80]	MS/MS	Identification of 240 *S*-nitrosylated proteins detected to address nitric oxide-induced capacitation
De Mateo et al. [81]	MALDI-TOF	Identification of 101 spots from infertile patients
Bohring et al. [22]	MALDI-MS	Identification of 18 proteins (sperm membrane antigens)

MALDI-TOF: matrix-assisted laser desorption/ionization-time of flight; LC-MS/MS: liquid chromatography-tandem mass spectrometry; LC-MS: liquid chromatography-mass spectrometry; MS/MS: tandem mass spectrometry.
